# Supplementary material for: Discrepancies between Promised and Actual AI Capabilities in the Continuous Vital Sign Monitoring of In-Hospital Patients: A Review of the Current Evidence
Source: Sensors (Basel). 2024 Oct 9;24(19):6497. doi: 10.3390/s24196497 (PMC11479359; doi:10.3390/s24196497)
Supplement: Supplementary file 1 [file sensors-24-06497-s001.zip › sensors-3216471-supplementary.pdf]

Sensors - Supplementary Material

Discrepancies Between Promised and Actual AI Capabilities in the Continuous Vital Sign Monitoring of In-Hospital Patients: A Review of the Current Evidence

Nikolaj Aagaard [1,*], Eske K. Aasvang [2,3,†] and Christian S. Meyhoff [1,3,†]

[1] Department of Anaesthesia and Intensive Care, Copenhagen University Hospital—Bispebjerg and
Frederiksberg, 2400 Copenhagen, Denmark; christian.sylvest.meyhoff@regionh.dk
[2] Department of Anaesthesia, Centre for Cancer and Organ Diseases, Copenhagen University Hospital—Rigshospitalet, 2100 Copenhagen, Denmark; eske.kvanner.aasvang.01@regionh.dk
[3] Department of Clinical Medicine, University of Copenhagen, 2200 Copenhagen, Denmark
* Correspondence: nikolaj.aagaard.01@regionh.dk; Tel.: +45-22908811
† These authors contributed equally to this work.

Supplemental Table S1. Alarm modification within the Visi Mobile System

Parameter	Delay (seconds)	Threshold (high/low)	Alarms/patient/day
Visi Mobile default alarms			
SpO_2	60	OFF / 85%	2.32
Heart rate	5	150 / 30	0.67
Respiratory rate	120	35 / 4	0.29
Continous blood pressure	SYS: 120<br>MAP: 90	SYS: 190/OFF<br>MAP: OFF/60	1.23
**Total**			**4.51**
Visi Mobile alarm optimization in case study			
SpO_2	*90*	OFF / 85%	*1.14*
Heart rate	*5*	150 / 30	*1.27*
Respiratory rate	*120*	35 / 4	*0.38*
Continous blood pressure	SYS: 240<br>MAP: 60	SYS: 200/OFF<br>MAP: OFF/58	*0.49*
**Total**			**3.29**

Supplemental Table S2. Algorithms used in the National Early Warning Score and the new multi-parameter real-time warning score

The NEWS							
Parameter	3	2	1	0	1	2	3
Respiratory rate (BrPM)	≤8		9-11*	12-20*		21-24*	≥25*
Oxygen saturation (%)	≤91	92-93	94-95	≥96			
Supplemental oxygen		yes		No			
Temperature (°C)	≤35		35.1-36	36.1-38	38.1-39	≥39.1	
Systolic bloodpressure (mmHg)	≤90	91-100	101-110	111-219			≥220
Heart rate (BPM)	≤40		41-50	51-90	91-110	111-130	≥131
Level of consciousness				A			V, P, U
A multi-parameter real-time warning score							
Parameter	3	2	1	0	1	2	3
Respiratory rate (BrPM)	≤8		9-11	12-20	21-24	25-30	≥30
Oxygen saturation (%)	≤91	92-93	94-95	≥96			
Temperature (°C)	≤35		35.1-36	36.1-38	38.1-39	39.1-40	≥40.1
Systolic blood pressure (mmHg)	≤90		91-100	101-140	141-180	181-200	≥221
Diastolic blood pressure (mmHg)	≤40	41-60		61-90		91-110	≥111
Heart rate (BPM)	≤40		41-50	51-90	91-110	111-149	≥150
Stroke volume (mL)	≤50		51-60	61-100	101-120		≥121
Cardiac output (L/minute)	≤2.5		2.6-4.0	4.1-8.0	8.1-12.0		≥12.1
Systemic vascular resistance (dynes*sec*cm^{-5})	≤600	601-800		801-1200	1201-1800	1801-2000	≥2001
Escalation for a multi-parameter real-time warning score							
Low	Up to one parameter deviating for 10 consecutive minutes (two consecutive measurements) with a score of 2						
Medium	Two parameters deviating simultaneously for 10 consecutive minutes (two consecutive measurements) both with a score of 2						
High	Three parameters or more deviating simultaneously for 10 consecutive minutes (two consecutive measurements) all with a score of 2						
Urgent	At least one parameter with a score of 3 for over 10 consecutive minutes (two consecutive measurements)						

*Numbers not provided in study
